# Nanostructure‐Dependent Signal Intensity in Through‐Hole Porous Alumina Membranes for Mass Spectrometry Imaging

**DOI:** 10.1002/rcm.10149

**Published:** 2025-09-30

**Authors:** Masahiro Kotani, Takashi Yanagishita

**Affiliations:** ^1^ Department of Applied Chemistry Tokyo Metropolitan University Hachioji Tokyo Japan

**Keywords:** imaging, MALDI, MSI, SALDI

## Abstract

**Rationale:**

Matrix‐assisted laser desorption/ionization (MALDI) is a widely used analytical technique for measuring high‐molecular‐weight compounds such as proteins. However, in the low‐molecular‐weight region, interference peaks derived from the matrix occur. Surface‐assisted laser desorption/ionization (SALDI), which is matrix‐free, does not generate background noise in the low‐molecular‐weight region and has the advantages of simple sample preparation and reproducibility. We previously developed an ionization method using an anodic porous alumina membrane (APAM) as a SALDI substrate. In this study, we examined the effects of the surface nanostructural properties of APAMs, such as hole diameter and pitch, on the signal intensity in mass spectrometry (MS) imaging.

**Methods:**

APAMs were fabricated using electrolytes of oxalic, malonic, and malic acids and were evaluated by MS using droplet samples and MS imaging. Droplet samples were applied to the back surface of the APAMs. MS imaging was conducted using 20‐μm‐thick mouse brain sections to compare the signal‐to‐noise ratio (SNR) of each APAM.

**Results:**

APAMs were fabricated with hole diameters (D_h_) of 24–419 nm, interhole distances (D_int_) of 100–625 nm, and open area ratios (OAR) of 5%–46%. From MS and MS imaging results, signal intensity at the same OAR increased in the order of D_int_ = 100, 625, and 270 nm, and the condition D_h_/D_int_ = 131/270 nm provided the highest SNR. In addition, APAMs with a D_h_ of less than 84 nm and an OAR lower than 10% exhibited lower signal intensities.

**Conclusions:**

We fabricated APAMs under various conditions and identified the processing conditions that provided the highest SNR of SALDI imaging. SALDI imaging using the APAMs fabricated under these high SNR conditions is expected to be applicable in various fields such as materials science and metabolomics, as it does not generate interference peaks in the low‐molecular‐weight region.

## 1 Introduction

Matrix‐assisted laser desorption/ionization (MALDI) is a widely used analytical technique for measuring high‐molecular‐weight compounds, and it is extensively utilized in the structural analysis of biomolecules and synthetic polymers. The ionization mechanism of the MALDI method involves mixing the sample with an organic compound matrix that absorbs ultraviolet (UV) light followed by irradiating the cocrystal with a UV laser to ionize the sample molecules [1, 2]. In recent years, many studies have focused on mass spectrometry (MS) imaging, where the matrix is applied to the sample and the laser is scanned to visualize the localization information of compounds [3, 4, 5, 6, 7, 8, 9, 10]. On the other hand, MALDI, which uses an organic matrix for sample preparation, generates interference peaks from the matrix in the low‐molecular‐weight region. In addition, the pretreatment process is complex owing to the need to select the optimal matrix and determine the appropriate conditions for the target substances. In MALDI imaging, reproducibility is an issue because of the variability in crystal formation after matrix spraying, and spatial resolution depends on the crystal size of the matrix. Although the matrix deposition method makes it possible to achieve uniform coating and small crystal sizes, it has problems such as the need for expensive equipment and low sensitivity.

Surface‐assisted laser desorption/ionization (SALDI) has been developed as a soft ionization method to solve these problems. SALDI methods are broadly categorized into two types: those that use nanostructured substrates and those that use inorganic particles for absorbing UV light instead of an organic matrix. Both methods desorb and ionize only the sample molecules. Compared with MALDI, matrix‐free SALDI does not generate interfering peaks in the low‐molecular‐weight region and has the advantages of easy sample preparation and better reproducibility. As a representative SALDI method using nanostructures, Sizudak et al. reported the desorption/ionization on silicon (DIOS) method, which uses porous silicon as the SALDI substrate prepared by electrochemical etching [11, 12, 13, 14]. DIOS is a simple pretreatment method that only requires dropping the measurement sample onto the substrate; however, the ionization capability decreases over time. This is due to the oxidation of the silicon surface caused by its reaction with oxygen in the air [15, 16]. In addition to silicon, other SALDI substrates such as porous alumina produced by the anodic oxidation of aluminum, titania nanotubes produced by the anodic oxidation of titanium, graphene, and carbon nanotubes have been reported [17, 18, 19, 20, 21, 22, 23, 24]. However, similarly to DIOS, methods using such substrates were difficult to apply to MS imaging. The reason is considered to be that when sample sections, which are thicker than the nanostructures, are placed on the SALDI substrate and irradiated with a laser, the SALDI effect is lost. The application of SALDI using silicon nanopost array (NAPA) structures for MS imaging was reported previously [25, 26, 27, 28]. However, it is necessary to reduce the thickness of the sample sections to less than 10 μm, which can be difficult depending on the type of tissue. On the other hand, SALDI methods using inorganic nanoparticles, such as metal nanoparticles (e.g., gold, platinum, and silver) and oxide nanoparticles (e.g., titanium oxide and iron oxide), have been reported [29, 30, 31, 32, 33, 34, 35]. These methods have also been applied to MS imaging. The pretreatment method is similar to that for MALDI. After preparing a suspension containing nanoparticles, the sample is either coated or mixed. In the case of metals, a deposition method can also be used. However, the coating process has the same issue of reproducibility as that for MALDI, and the deposition process also has the issues of reproducibility and variability because film thickness control is very stringent.

We previously investigated an ionization method using a porous alumina membrane with an array of submicron‐order through‐holes fabricated by an aluminum anodization process as a SALDI substrate [36, 37, 38, 39]. This anodic porous alumina membrane (APAM) has cylindrical nanopores and becomes chemically stable when crystallized by heat treatment [40]. In addition, the APAM can also be applied to MS imaging by utilizing through‐holes. MS imaging can be achieved by placing the APAM on a frozen sample section, thawing the sample to allow the sample components to be suctioned through capillary action while maintaining their spatial information, and then irradiating the APAM surface with a laser. The spatial resolution limit of MS imaging is determined by the hole pitch (interhole distance) of the APAM, and submicron resolution can be achieved in principle when the laser beam diameter is sufficiently reduced. The hole diameter, pitch, and open area ratio (OAR) of APAMs can be easily controlled by the electrolyte and electrolysis conditions, and the film thickness can be freely changed by adjusting the treatment time. A previous study has shown that the nanostructure of APAMs affects the MS signal intensity of droplet samples [39]. Our research aims to systematically analyze how this nanostructure influences the sensitivity of APAMs when used as a SALDI substrate for MS imaging. An MS imaging method with low background noise in the low‐molecular‐weight region is crucial for visualizing trace components in fields such as food science, industrial materials, cosmetics, and plant biology. Since APAMs theoretically provide spatial resolution equivalent to their hole pitch, they enable single‐cell level MS imaging for the analysis of metabolites and other cellular compounds. However, in LDI‐MS imaging, pursuing high spatial resolution leads to a necessary trade‐off with sensitivity. This is because a smaller laser spot size inevitably reduces the amount of detected ions. APAMs also suffer from a decrease in sensitivity with high spatial resolution, making the high‐sensitivity APAM proposed in this study indispensable. The APAM is expected to contribute to the ultra‐trace analysis of biomolecules in medical and pharmaceutical fields through single‐cell level MS imaging.

The nanostructure of APAMs is thought to significantly affect ion generation efficiency, as the MS imaging technique suctions sample components from the back surface via capillary action. The hole diameter is associated with the ease of suction of sample components, while the OAR is related to the amount of sample components suctioned onto the surface. Previous studies have suggested that even with the same OAR, variations in interhole distance can affect signal intensity [39]. Therefore, in this study, we systematically investigated the optimization of hole diameter, interhole distance, and OAR.

## 2 Methods

### 2.1 Preparation of APAMs

**FIGURE 1:**
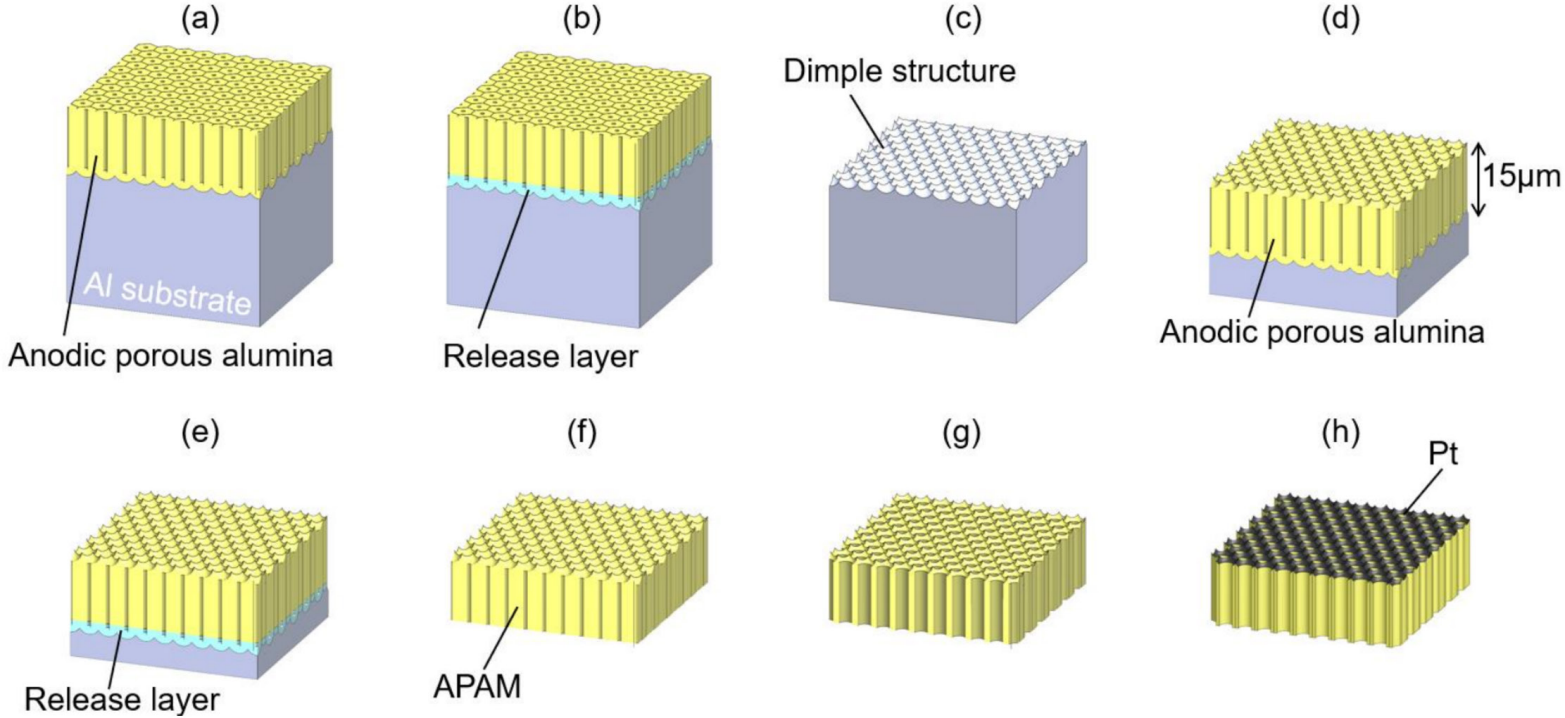
Schematic of the fabrication process for through‐hole anodic porous alumina membranes (APAMs): (a) first anodization, (b) formation of release layer by anodizing in 17.6‐M sulfuric acid, (c) removal of release layer, (d) second anodization, (e) formation of the release layer by anodizing in 17.6‐M sulfuric acid, (f) APAMs obtained after removing the release layer, (g) etching to enlarge the hole diameter, and (h) Pt coating on APAMs.

Figure 1 shows the schematic of the fabrication process for APAMs. A 99.99% pure aluminum plate (Nippon Light Metal Co. Ltd.) was ultrasonically degreased in acetone (FUJIFILM Wako Pure Chemical Corporation) for 5 min, followed by chemical polishing at 120°C for 2 min using a mixture of 68 wt% phosphoric acid (Kanto Chemical Co. Inc.) and 3 wt% nitric acid (FUJIFILM Wako Pure Chemical Corporation) to smooth the surface. APAMs were fabricated using a two‐step anodization method to improve the regularity of pore arrangement. Previous studies have shown that the interpore distance of APAMs is proportional to the processing voltage and can be controlled by adjusting the voltage [41]. The first anodization was carried out using electrolytes of oxalic acid (FUJIFILM Wako Pure Chemical Corporation), malonic acid (FUJIFILM Wako Pure Chemical Corporation), and malic acid (FUJIFILM Wako Pure Chemical Corporation) under the anodization conditions shown in Table 1 (Figure 1a). A small amount of phosphoric acid was mixed with the malic acid electrolyte to improve the self‐ordering of the pore arrangement [42]. The anodization time was adjusted to obtain a porous alumina film thickness of 15 μm. For stable analysis in MS imaging using APAMs, a membrane thickness of at least 15 μm was required to prevent cracking. However, excessive membrane thickness can result in the diffusion of laser‐induced heat into the substrate, thereby reducing the efficiency of desorption and ionization. Accordingly, a thickness of 15 μm was selected as the optimal value to ensure mechanical robustness while maintaining high signal intensity. To remove the first‐anodization film, a release layer was formed by anodizing in a 17.6‐M sulfuric acid (FUJIFILM Wako Pure Chemical Corporation) electrolyte at 0°C for 60 min (Figure 1b). The processing voltages were the same as those in the first anodization, namely, 40, 110, and 250 V. This release layer incorporates large amounts of sulfate anions inside the anodized film, making it possible to selectively dissolve and remove it using a low‐concentration acid [43]. The release layer formed by anodizing in high‐concentration sulfuric acid was dissolved and removed with 1 wt% phosphoric acid, resulting in a dimple structure on the surface of the Al plate (Figure 1c). The Al plate was re‐anodized under the same conditions as those in the first anodization (Figure 1d). Following the same process as in Figure 1b,c, APAMs were obtained by forming and removing the release layer (Figure 1e,f). The hole size of the obtained APAMs was enlarged using 10 wt% phosphoric acid at 30°C for the etching times shown in Table 1 (Figure 1g). The upper limit of the etching time was determined based on the membrane strength required to prevent cracking during handling in MS imaging and to ensure stable MS evaluation. The malonic acid anodization at 110 V resulted in a lower OAR and a more random hole diameter and arrangement compared to other conditions. This is considered to have increased the number of interconnected adjacent holes as etching progressed, leading to a decrease in physical membrane strength. A 50‐nm Pt layer was deposited on the surface of the APAM, forming a SALDI substrate (Figure 1h). Pt was deposited to accelerate ions generated by laser irradiation through an electric field and introduce them into the mass separation section. The surface of the APAM was observed by scanning electron microscopy (SEM, JSM‐IT500HR, JEOL), followed by binarization processing to calculate the average hole diameter and OAR from the area of each hole.

**TABLE 1:** Conditions for each anodization and etching treatment.

Electrolyte	Voltage (V)	Temperature (°C)	Anodization time (min)	Etching time (min)
0.3‐M oxalic acid	40	16	130	0, 10, 20
1.0‐M malonic acid	110	15	120	0, 20, 40
0.2‐M malic acid + 2.0‐mM phosphoric acid	250	30	540	0, 60, 120

### 2.2 MS

Figure S1 shows the measurement procedures for (a) droplet samples and (b) MS imaging of mouse brain sections. For (a), 1 μL of a droplet sample was dropped onto the back surfaces of APAMs. After the sample was dried, the APAMs were turned over and fixed to a slide glass with Al tape. The APAMs were measured by MALDI‐TOF MS (autoflex, Bruker). To leverage the matrix‐free characteristics of APAMs, a range of droplet samples with varying molecular weights was selected. This range extends from the low‐molecular‐weight region, which is susceptible to interference from matrix‐derived background noise, to peptides with potential applications in single‐cell MS imaging. Due to the potential application of APAM‐based MS imaging in food science and industrial materials, sucrose (FUJIFILM Wako Pure Chemical Corporation) was selected as a representative water‐soluble organic compound of biological origin for droplet samples. Cesium iodide (CsI, FUJIFILM Wako Pure Chemical Corporation) was chosen as an inorganic model compound. Furthermore, considering the potential of high spatial resolution for single‐cell MS imaging, angiotensin II (FUJIFILM Wako Pure Chemical Corporation) was selected as a representative biological macromolecule. Angiotensin II is an important mid‐sized molecule often studied as a drug or physiologically active substance. Each sample was adjusted to concentrations of 1, 50, and 10 μM using pure water. Angiotensin II was mixed with an equal amount of a proton donor, which was prepared by dissolving 5‐mg mL^−1^ citric acid (FUJIFILM Wako Pure Chemical Corporation) and 5‐mg mL^−1^ diammonium hydrogen citrate (FUJIFILM Wako Pure Chemical Corporation) in pure water and mixing them. The laser conditions were set to a random walk with 500 accumulated shots, and the measurements were conducted in the positive ion, reflectron mode, with the same laser power applied to each sample. The acquired mass spectra were analyzed using the flexAnalysis software (Bruker). The measurement procedure for (b) MS imaging involved setting a mouse brain section onto a slide glass, and an APAM was placed on top. The mouse brain section was thawed by warming the back of the slide glass with the heat of a finger, and the sample components were suctioned from the back of the APAM by capillary action. After drying the sample, the periphery of the APAM was fixed with Al tape and measured by MALDI‐TOF MS. The brains of 8‐week‐old male ICR mice (Japan SLC Inc.) were used, which were sectioned to a thickness of 20 μm using a cryostat (CM3050S, Leica Biosystems). All serial sections used for MS imaging were prepared on the same day from a single mouse brain using a cryostat. After sectioning, samples that had completed the suction process using APAMs were placed in a sealed container with silica gel and stored at −80°C to minimize the effects of moisture and chemical changes until measurement. To eliminate variability in measurement conditions during MS imaging, the samples were returned to room temperature immediately before analysis. They were then removed from their sealed container and promptly introduced into the MALDI‐TOF MS. MS imaging was conducted in the positive ion, reflectron mode, accumulating 200 shots at each point. The laser pitch and beam diameter were both set to 50 μm. The data obtained from MS imaging were analyzed using the flexImaging software (Bruker).

## 3 Results and Discussion

### 3.1 Fabrication of APAMs

Figure S2 shows an optical image of the APAM appearance. The APAM became warped after fabrication because of the rolling of the aluminum plate. The warping of the membrane causes the laser focus to shift during MS, resulting in variation in signal intensity across the surface. To suppress this variation, Al tape was attached around APAMs. The effective area of the APAM was 25 × 18 mm.

**FIGURE 2:**
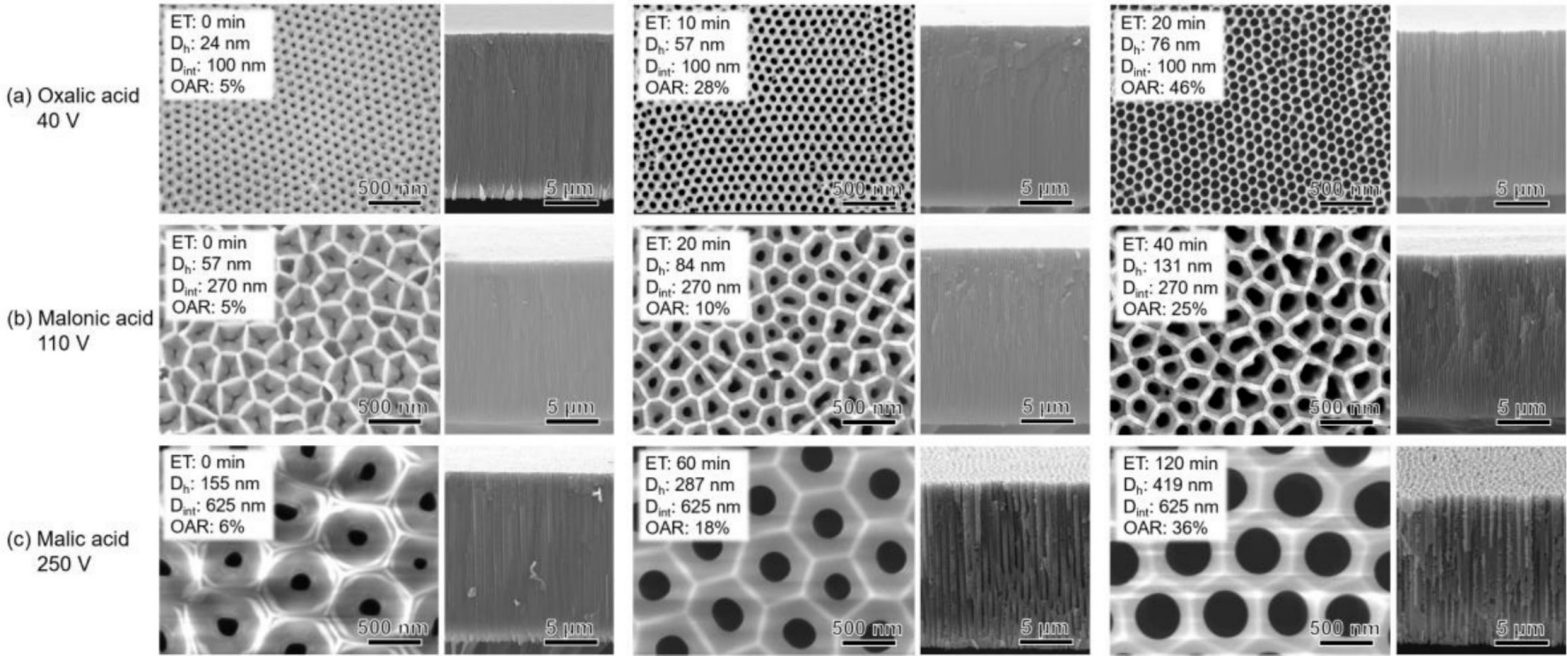
Surface and cross‐section SEM images of APAMs anodized with (a) oxalic acid at 40 V, (b) malonic acid at 110 V, and (c) malic acid at 250 V.

Figure 2 shows SEM images of APAMs with different interhole distances and hole diameters. The APAMs were fabricated using (a) oxalic acid at 40 V, (b) malonic acid at 110 V, and (c) malic acid at 250 V. The SEM images show the etching time (ET), hole diameter (D_h_), interhole distance (D_int_), and calculated OAR. SEM images confirmed that the interhole distance increased proportionally with the anodization voltage, which was generally consistent with the relationship found in previous studies: D_int_ = 2.5 × anodizing voltage [44]. In addition, the hole size was controlled by etching within the ranges of D_h_ = 24–76 nm (OAR, 5%–46%) for oxalic acid at 40 V, D_h_ = 57–131 nm (OAR, 5%–25%) for malonic acid at 110 V, and D_h_ = 155–419 nm (OAR, 6%–36%) for malic acid at 250 V.

### 3.2 Mass Spectrometry Results

**FIGURE 3:**
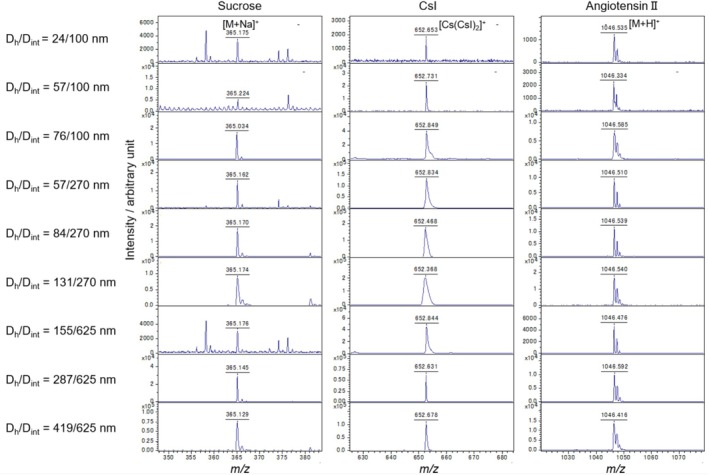
Mass spectra obtained from droplet samples of 1‐mM sucrose, 50‐mM CsI, and 10‐μM angiotensin II by using APAMs of various nanostructures.

Figure 3 shows the mass spectra of sucrose, CsI, and angiotensin II under each anodization condition. The signal of 1‐mM sucrose was confirmed to be the sodium adduct [M + Na]^+^ in all APAMs. Because no cationizing agent was mixed with the sucrose used for the measurement, the adducted sodium is considered to have reacted with sucrose molecules from the atmosphere, from equipment such as pipette tips and microtubes, or from sodium attached during the APAM fabrication process. In the mass spectra of CsI, several cluster ions formed through the combination of CsI molecules and Cs ions were detected. However, in this study, we focused on comparing the signal of [Cs (CsI)_2_]^+^. In 50‐mM CsI, all APAMs detected the signal of [Cs (CsI)_2_]^+^. The broadened peak widths of D_h_/D_int_ = 76/100 and 57–131/270 nm are considered attributable to the spatial spreading of ions due to excessively high laser power. Reducing the laser power would result in sharper peak widths. However, it was necessary to compare signal intensities using the same laser power in this experiment. Consequently, the high signal‐to‐noise ratio (SNR) APAMs exhibited broadened peak widths. By mixing 10‐μM angiotensin II with an equal amount of citric acid as a proton donor, we confirmed the proton adduct [M + H]^+^ signal in all APAMs. For all droplet samples, the condition of D_h_/D_int_ = 131/270 nm provided the highest signal intensity, whereas D_h_/D_int_ = 24/100 nm provided the lowest signal intensity.

**FIGURE 4:**
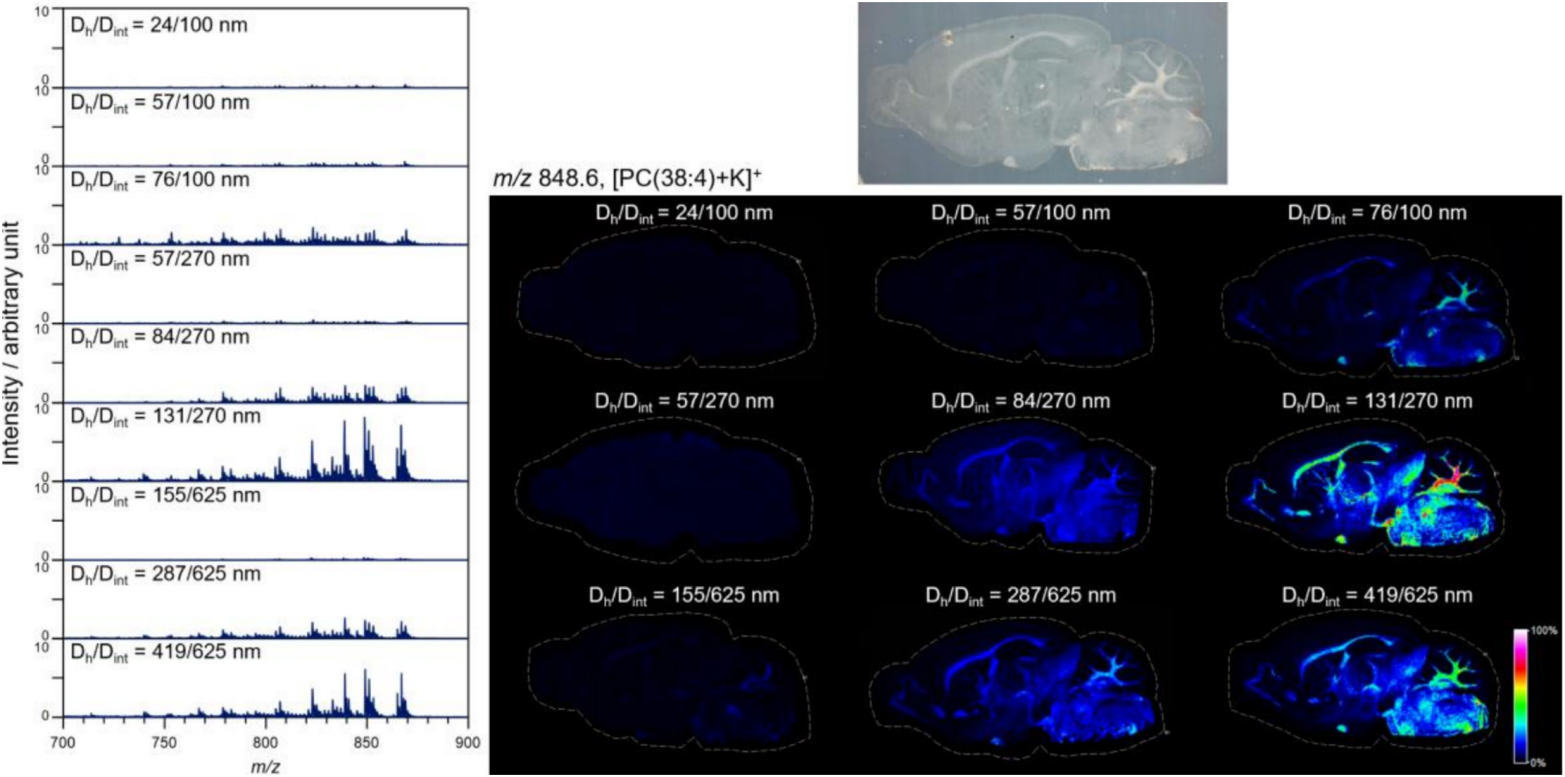
Average mass spectra covering *m/z* range of phospholipids and MS images at *m/z* 848.6 [PC(38:4) + K]^+^ obtained from mouse brain sections by using APAMs of various nanostructures.

Figure S3 shows the average mass spectrum (*m/z* 100–1000) obtained by MS imaging of mouse brains for each APAM, with baseline correction applied using the sliding window method. Figure 4 shows the average mass spectra of phospholipids at *m/z* 700–900. The optical image shows a transmission view of a mouse brain section and the APAM placed on a slide glass. The ion images show heat maps at *m/z* 848.6, [PC(38:4) + K]^+^, and the heat maps were created using relative values with that for the pixel having the highest signal intensity among all MS images set as 100%. The average mass spectra and ion images suggest that signals can hardly be detected at D_h_ of less than 57 nm, regardless of the interhole distance. Therefore, D_h_ = 57 nm or larger is required to suction the sample components of the mouse brain by capillary action. Similar to the droplet samples, the condition D_h_/D_int_ = 131/270 nm exhibited the highest signal intensity, whereas the condition D_h_/D_int_ = 24/100 nm showed the lowest signal intensity.

**FIGURE 5:**
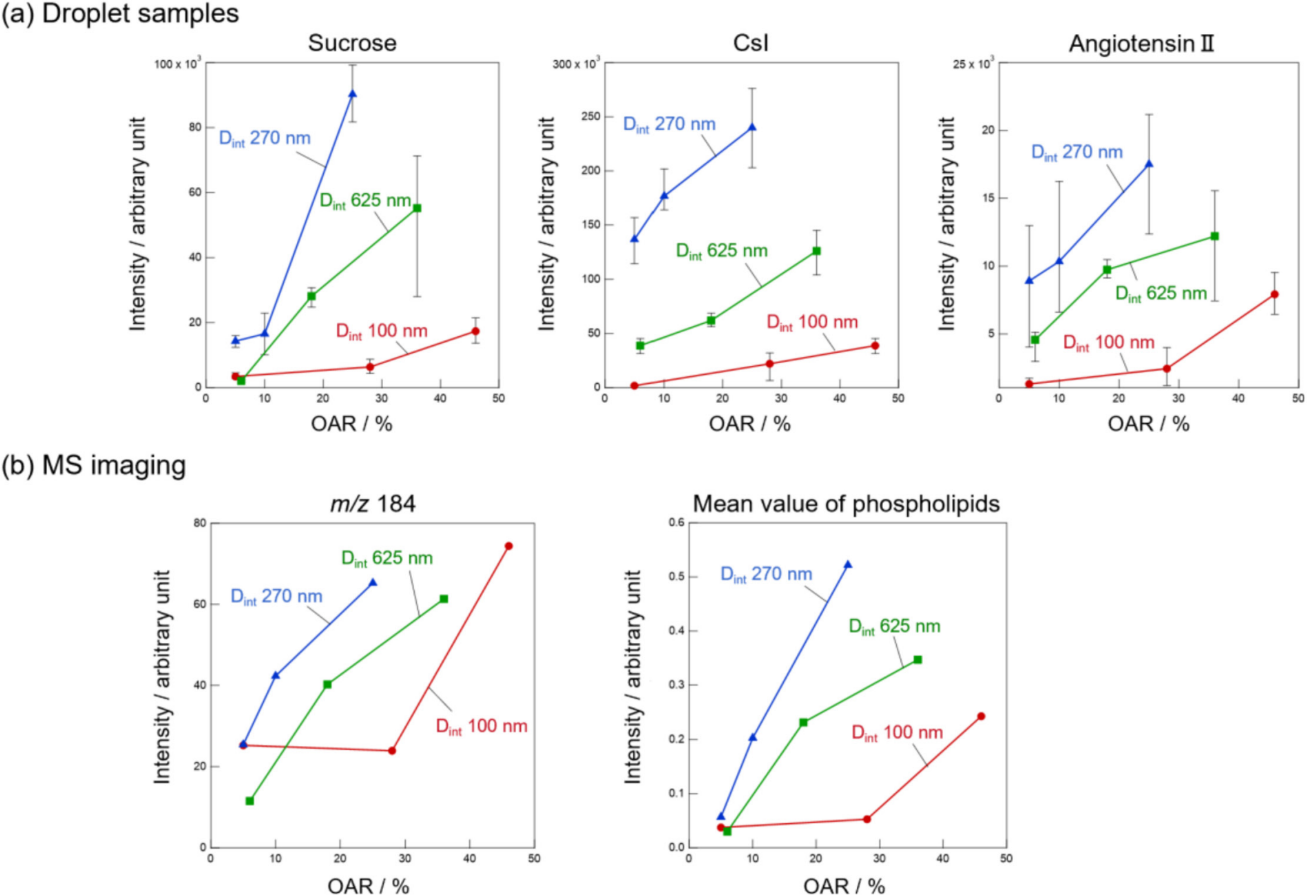
Relationship between signal intensity from (a) droplet samples and (b) MS imaging and OAR of each APAM.

We investigated the correlation between the MS signal intensity and the nanostructure of APAMs in detail. Figure 5 shows the relationship between the OAR and signal intensity of APAMs from the results of (a) droplet samples and (b) MS imaging. The error bars shown in the graph represent the minimum and maximum values of the data obtained from multiple measurements, while the plotted values are the average of these measurements. (b) MS imaging shows the relationships of the fragment ion *m/z* 184, [C_5_H_15_NO_4_P]^+^ derived from the phospholipid head group with the average intensity of phospholipids *m/z* 700–900 and OAR. Since *m/z* 184 is the signal intensity of a fragment ion, when the signal intensity of phospholipids is the same, a lower signal intensity of *m/z* 184 indicates softer ionization. From the results of both (a) droplet samples and (b) MS imaging, the signal intensity at the same OAR increased in the order of D_int_ = 100, 625, and 270 nm, and the condition D_h_/D_int_ = 131/270 nm exhibited the highest SNR, similar to the results of the droplet samples. In addition, focusing on the same interhole distance, an increase in the OAR of APAMs resulted in higher signal intensity. Increasing the amount of sample molecules on the laser irradiation surface is also important for achieving high signal intensity.

The low SNR observed in the APAM with a small OAR is considered to be due to the reduced absolute amount of sample components suctioned by capillary action, resulting in fewer sample molecules reaching the laser irradiation surface. Although the OAR of D_h_/D_int_ = 76/100 nm was the largest, the signal intensity was not particularly high. This is considered to be due to the fact that fragmentation occurred most extensively under the conditions shown in the graph for *m/z* 184. Furthermore, D_h_/D_int_ = 131/270 nm exhibited higher signal intensity despite having a lower OAR than D_h_/D_int_ = 419/625 nm. This could be attributed to the effect of the anion content within the anodized film. Previous studies have reported that anodization at high current densities results in increased incorporation of electrolyte‐derived anions into the oxide film [45]. According to Table 1, the anodization time required to fabricate APAMs with D_int_ = 270 nm is shorter than that for D_int_ = 625 nm. This indicates that the current density is higher, suggesting increased incorporation of electrolyte‐derived anions into the oxide film. Vera‐Londono et al. have suggested that anodic oxide films containing a higher concentration of electrolyte‐derived anions exhibit reduced thermal conductivity [46]. Such a low thermal conductivity film facilitates localized heat accumulation upon laser irradiation. This promotes efficient vaporization and ionization of sample molecules, which is considered to contribute to the enhancement of signal intensity. Although anions within the APAM may also vaporize upon laser irradiation, the amount of anions released from the oxide film is considered to be significantly lower than that of sample molecules. As a result, the reduction in cation signals due to neutralization in the gas phase was likely limited. Based on the above, the result that APAM with D_int_ = 270 nm exhibited higher SNR than that with D_int_ = 625 nm is presumed to be primarily due to enhanced ionization efficiency, which is attributed to differences in film structure and thermal conductivity.

Figure 5 shows that APAMs with Dint = 57 nm or smaller exhibited low MS signal intensity regardless of their OAR. The two main causes of this are thought to be a decrease in suction efficiency due to capillary action and the physical confinement of sample components during laser ablation. The equation for capillary action is given by:

$$h=\frac{2\text{Tcosθ}}{\mathrm{\rho gr}}$$

where *h* is the height of a liquid column, *T* is the liquid‐air surface tension, *θ* is the contact angle, *ρ* is the density of liquid, *g* is the gravity acceleration, and *r* is the tube radius [47]. From the above equation, the height of the water surface raised by capillary action increases as the pore diameter decreases. Vo et al. reported that in extremely fine holes with diameters of 2 nm or smaller, intermolecular interactions with the hole walls lead to a localized increase in liquid viscosity, resulting in a reduced rate of capillary suction [48]. While the hole diameters in this study are larger than those reported by Vo et al., considering the unique fluid behavior at the nanoscale, the possibility of a partial decrease in suction efficiency due to some viscous effects cannot be ruled out. However, the more dominant factor is presumed to be the physical confinement of sample components. As shown by Chichkov et al. regarding the interaction between laser ablation and nanostructures [49], this study also suggests that sample components introduced into the nanochannels via capillary action were physically confined within the narrow holes. As a result, efficient desorption and ionization by laser irradiation became difficult, leading to a decrease in the number of ions reaching the mass spectrometer detector and consequently causing a reduction in signal intensity.

## 4 Conclusion

In this study, we investigated the conditions that provided high SNR of APAMs fabricated by anodizing aluminum as a SALDI substrate. The APAMs were anodized with oxalic acid at 40 V, malonic acid at 110 V, and malic acid at 250 V, resulting in D_h_ = 24–419 nm, D_int_ = 100–625 nm, and OAR = 5%–46%. From the results of sucrose, CsI, and angiotensin II dropped on the back surface of each APAM, as well as the MS imaging results of the mouse brain, the signal intensity at the same OAR increased in the order of D_int_ = 100, 625, and 270 nm. The condition of D_h_/D_int_ = 131/270 nm provided the highest SNR. At the same interhole distance, APAMs with higher OAR exhibited increased signal intensity. APAMs with a small OAR are considered to have low SNR because of the small amounts of sample components suctioned by capillary action. In addition, the reduced signal intensity observed in APAMs with D_h_ = 57 nm or smaller is attributed to the physical confinement of sample components within narrow holes introduced via capillary action, which hindered efficient desorption and ionization. In this study, the SNR was confirmed to be determined by a complex interplay of multiple factors, including not only the hole diameter but also the OAR and anions incorporated into the alumina substrate. SALDI imaging using the APAM fabricated under the conditions that provided high sensitivity is expected to have applications in various fields such as materials science and metabolomics.

## Author Contributions

**Masahiro Kotani:** validation, conceptualization, data curation, methodology, investigation, visualization, writing – original draft. **Takashi Yanagishita:** supervision, funding acquisition, writing – review and editing, validation, project administration, resources, conceptualization.

## Peer Review

The peer review history for this article is available at https://www.webofscience.com/api/gateway/wos/peer‐review/10.1002/rcm.10149.

## Supporting information


**Figure S1:** Measurement procedure for (a) droplet sample: dropping onto the back surface of APAMs, drying the sample, and turning the APAMs upside down and fixing with Al tape. Measurement procedure for (b) MS imaging of mouse brain using APAMs: placing APAMs on a frozen mouse brain section, thawing with finger heat and suction of the sample components by capillary action, and fixing with Al tape.
**Figure S2:** Optical image of APAMs after Al tape bonding.
**Figure S3:** Average mass spectra (*m/z* 100–1000) from MS imaging using APAMs of various nanostructures.

## Data Availability

The data that support the findings of this study are available from the corresponding author upon reasonable request.
