# Supplementary material for: Nanostructure‐Dependent Signal Intensity in Through‐Hole Porous Alumina Membranes for Mass Spectrometry Imaging
Source: Rapid Commun Mass Spectrom. 2025 Sep 30;40(1):e10149. doi: 10.1002/rcm.10149 (PMC12514746; doi:10.1002/rcm.10149)
Supplement: Supplementary file 1 — Figure S1: Measurement procedure for (a) droplet sample: dropping onto the back surface of APAMs, drying the sample, and turning the APAMs upside down and fixing with Al tape. Measurement procedure for (b) MS imaging of mouse brain using APAMs: placing APAMs on a frozen mouse brain section, thawing with finger heat and suction of the sample components by capillary action, and fixing with Al tape. Figure S2: Optical image of APAMs after Al tape bonding. Figure S3: Average mass spectra (m/z 100–1000) from MS imaging using APAMs of various nanostructures. [file RCM-40-e10149-s001.docx]

**Nanostructure-dependent signal intensity in through-hole porous alumina membranes for mass spectrometry imaging**

Masahiro Kotani, Takashi Yanagishita*

Department of Applied Chemistry, Tokyo Metropolitan University, 1-1 Minamiosawa, Hachioji, Tokyo 192-0397, Japan

*^*^E*-*mail: yanagish@tmu.ac.jp*

Abstract

Contents

- Figure S1. Measurement procedure using APAMs for (a) droplet sample and (b) MS imaging of mouse brain.
- Figure S2. Optical image of APAMs
- Figure S3. Average mass spectra from MS imaging using APAMs of various nanostructures.


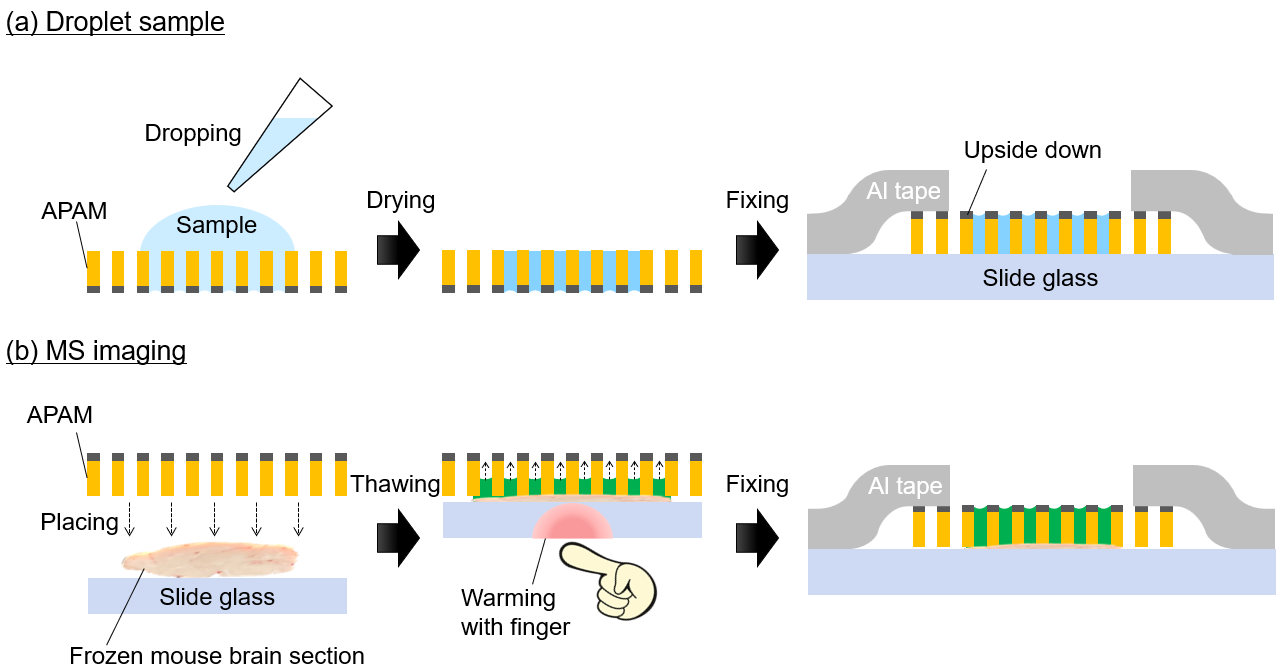


**Figure S1.** Measurement procedure for (a) droplet sample: dropping onto the back surface of APAMs, drying the sample, and turning the APAMs upside down and fixing with Al tape. Measurement procedure for (b) MS imaging of mouse brain using APAMs: placing APAMs on a frozen mouse brain section, thawing with finger heat and suction of the sample components by capillary action, and fixing with Al tape.


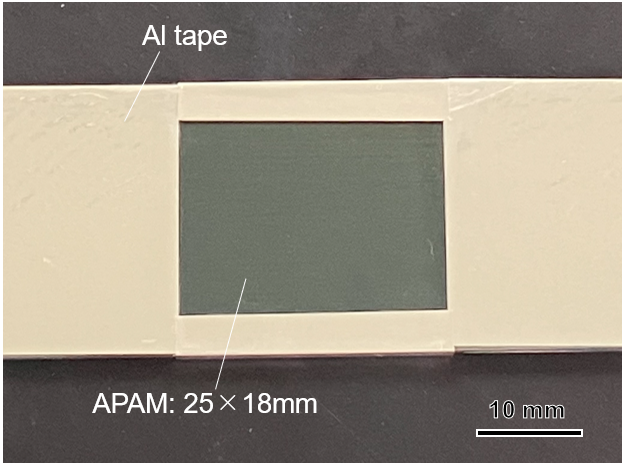


**Figure S2.** Optical image of APAMs after Al tape bonding.


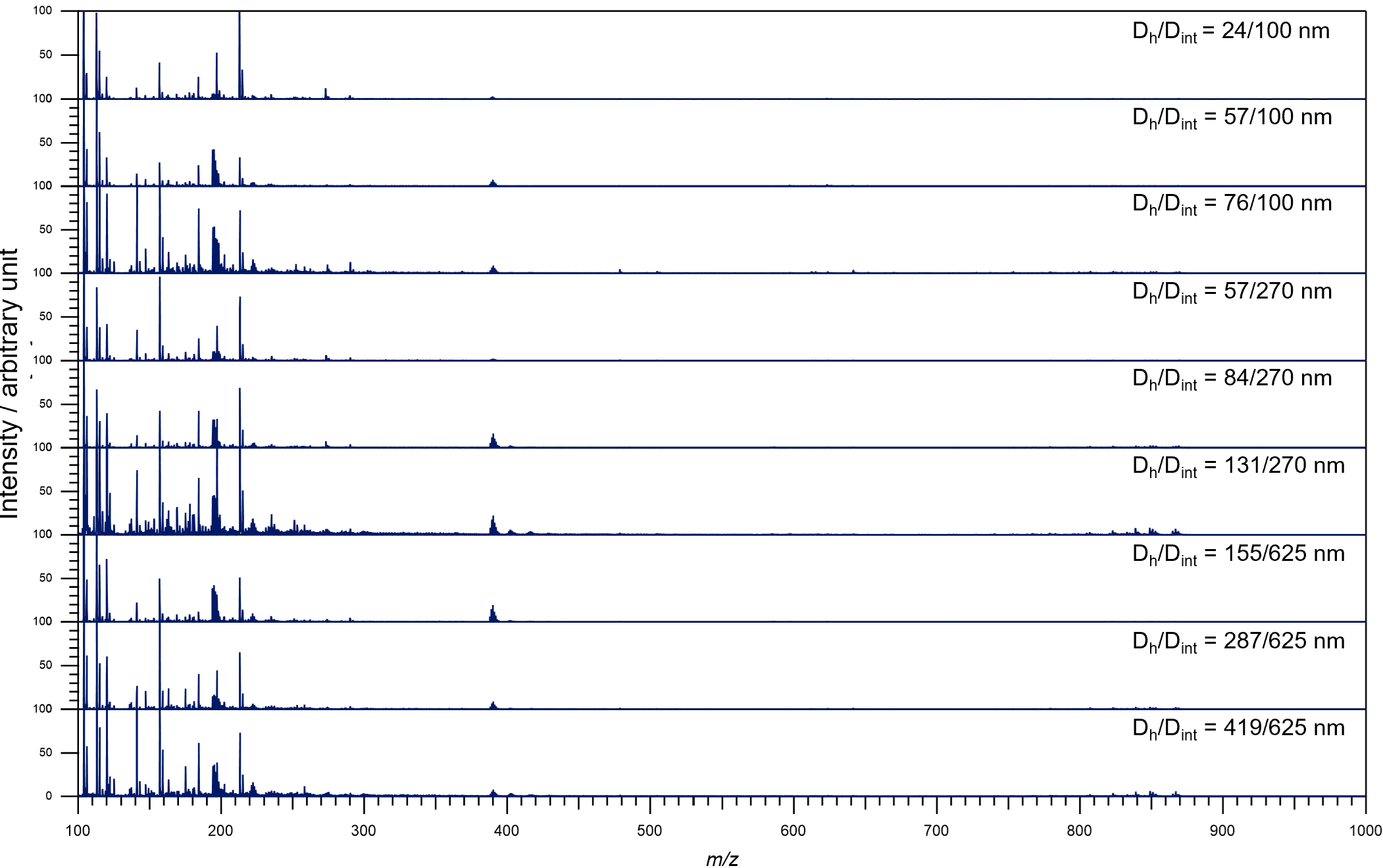


**Figure S3.** Average mass spectra (*m/z* 100–1000) from MS imaging using APAMs of various nanostructures.
